# Blessing or curse: the role of authoritarian filial piety and self-efficacy in caregiver gains among Chinese family caregivers caring for physically impaired older adults

**DOI:** 10.1186/s12877-024-04768-x

**Published:** 2024-02-16

**Authors:** Jiyuan Zhang, Xin Sun, Zi Yan

**Affiliations:** 1 School of Public Administration, East China Normal University, Shanghai, 200062 China; 2 School of Social Development and Public Policy, Fudan University, Shanghai, 200433 China; 3 Waseda Institute for Advanced Studies, Waseda University, Tokyo, 169-8050 Japan

**Keywords:** Caregiver burden, Caregiver gains, Caregiver self-efficacy, Filial piety, Moderated mediation effect, China

## Abstract

**Background:**

This study investigated the effects of authoritarian filial piety (AFP) and caregiver self-efficacy on the caregiving experience of adult children of physically impaired older adults. Socio-cultural stress and coping model was applied to test the influence of AFP on caregiver gains.

**Methods:**

A total of 601 Chinese adult children caregivers and care-recipient dyads participated in this cross-sectional study in 2021. Four instruments were used to collect data: the 4-item Zarit Burden Interview, Positive Aspects of Caregiving Scale, Caregiver Task Inventory Scale, and Authoritarian Filial Piety Scale. All mediation and moderated mediation effects were estimated using SPSS 26.0.

**Results:**

Caregiver self-efficacy was found to not only mediate but also help family caregivers convert their burden into positive gains. AFP moderates the association between caregiver burden and self-efficacy, as well as between caregiver burden and caregiver gains.

**Conclusions:**

This study provides valuable insights into filial piety, elucidating AFP’s comprehensive impact on cognitive appraisals of caregiving. Culturally sensitive psychoeducational therapy, addressing AFP expectations and boosting caregiver self-efficacy, is recommended to enhance positive caregiving outcomes.

**Supplementary Information:**

The online version contains supplementary material available at 10.1186/s12877-024-04768-x.

## Introduction

As life expectancy increases globally, populations age, demographics change, and family structures evolve, the challenge of providing care for frail older people and adults living with chronic physical and mental diseases becomes increasingly significant around the globe. It has been widely acknowledged that caregiving is a stressful activity that can result in an imbalance of care demands relative to caregivers’ time, social roles, physical and emotional states, and financial resources [1–4]. Family caregivers, also known as informal caregivers, are unpaid individuals providing essential assistance with daily living activities for dependent family members, encompassing elders, spouses, and children [5]. They constitute the primary pillar of society’s care support [6]. In light of the unprecedented global aging trend and declining fertility rates, mitigating the burden of caregiving holds substantial public health implications.

Caregiving is a complex process, and many factors influence caregivers’ coping mechanisms and adaptations to burdens and stress. Over the past few decades, the primary thrust of family caregiver research has focused mainly on the negative aspects of the process (for example, depression, stress, and role strain) [2, 3, 7, 8]. Although the burden is an important concern, other aspects of caregiving, including positive aspects [9–16] and the social and cultural values underpinning caregiver motivation [2, 17–20], also bear important implications for theory, research, policy, and practice. Theoretical frameworks such as the caregiver adaptation model [21], and the two-factor model of caregiving appraisal [22, 23] integrate both the unfavourable and favourable dimensions of caregiving, proposing that caregivers might experience emotional or cognitive advantages from their caregiving role [24]. It is noteworthy that, in the above two models, caregiver self-efficacy was identified as a mediator on the relationship between caregiver burden and caregiver gain [21, 25–27].

Sole consideration of caregiver self-efficacy does not provide a complete picture. Existing research has also documented that social and cultural values (e.g., familism and filial piety) strongly underpin the motivations and meanings of informal caregiving. The socio-cultural stress and coping model highlights the interplay between social and cultural factors in influencing stress and coping mechanisms [18, 25, 28]. For instance, a systematic review found that filial piety is an important element of the caregiving process (burden, coping, seeking information, and support) in many cultures [20], suggesting that it may be a critical component influencing caregiver self-efficacy.

In China, because of the Confucian cultural value of filial piety and the lack of a well-established home-and-community-based care system, adult children play a central role in providing long-term care for older parents. According to the dual filial piety model, reciprocal filial piety (RFP) and authoritarian filial piety (AFP) have been identified as two of the most important aspects of filial piety in Chinese societies [29]. RFP refers to gratitude and care for parents and is based on the principles of love, intimacy, and reciprocal relationships, reflecting interpersonal relatedness and one’s attitude. Previous studies in China have emphasized the significant influence of cultural values on caregiving experiences [19, 25, 30]. For instance, one study found that adult children caregiver’s burden/gains are related to their RFP [29]. By contrast, AFP emphasises sacrificing one’s own physical and psychological needs to provide care and support for one’s parents, glorify one’s parents, and continue the family lineage. Empirical findings suggest that AFP is more likely to be correlated with personal stress than RFP, as it often involves self-suppression and self-sacrifice [31]. AFP is also associated with maladaptation of intrapersonal stress, including lower self-efficacy, lower cognitive flexibility, and elevated prevalence of anxiety and depression [32]. These empirical studies have expanded our understanding of filial piety, elucidating its comprehensive impact on contemporary Chinese family dynamics, encompassing both positive and negative aspects. Nonetheless, to the best of our knowledge, few studies have investigated the role of AFP in the relationship between caregiver burden and gain.

Based on the socio-cultural stress and coping model, the present study supplements the existing research by investigating the impacts of caregiver self-efficacy and AFP on caregiver burden/gains among Chinese family caregivers. A total of 601 adult children caregiver/care-recipient dyads from four capital cities in the Yangtze River Delta region (Shanghai, Nanjing, Hangzhou, and Hefei) were recruited from July to August 2021. These cities not only exhibit aging rates surpassing the national average of 13.52% (Shanghai-16.28%, Jiangsu-16.20%, Zhejiang-13.27%, and Anhui-15.01%), but also display a heightened societal openness to social change, facilitating the examination and reconsideration of the effects of traditional AFP on caregiver burden/gains.

## Literature review and research hypothesis

### Caregiver burden and caregiver gains

Caregiver burden, also known as caregiver stress or caregiver burnout, is the extent of multifaceted stress (emotional, physical health, psychological social life, financial status) perceived by the caregiver arising from the sustained provision of care for a family member and/or loved one. It is divided into subjective burden and objective burden [33]. Caregiver burden is related to the well-being of both the caregiver and care-recipient. The Zarit Burden Interview (ZBI) [34] and Caregiver Burden Inventory (CBI) [33] are among the most common tools used to measure caregiver burden.

Caregiver gains, often referred to as positive aspects of caregiving (PAC), encompass positive caregiving appraisals. They are as genuine and integral to the caregiving journey as the burden and responsibility. While there is no consistent definition of PAC owing to various social contexts, empirical research on these aspects has highlighted the positive impact on psychological well-being within caregiving experiences, such as role satisfaction, emotional rewards, personal growth, faith/spiritual growth, relationship gains, a sense of duty, and reciprocity [10, 12, 14, 16]. A Chinese version of PAC scale has been developed and validated in previous studies [35]. The PAC scale has been developed for use in Chinese contexts, with multiple versions observed in prior studies.

### Caregiver self-efficacy

Defined as an individual’s belief in their capability to organise and execute necessary actions to achieve a goal [36], caregiver self-efficacy offers a comprehensive insight into how effectively a caregiver adapts and copes with stress/burden in caregiving [37]. Derived from the caregiver adaptation model [21], self-efficacy mediates the relationship between burden and gains in coping models by improving problem-solving capacity, improving self-esteem, and fostering caregiving resilience [25]. However, the role of self-efficacy in caregiving consequences has been inconsistent, particularly when caregiving gains are considered an outcome variable. Some studies have found that higher caregiver self-efficacy is advantageous for overall caregiving outcomes [26, 27, 38, 39], while other studies revealed that self-efficacy may not contribute to caregiver gains [25]. This leads to our first hypothesis.

- Hypothesis 1 (**H1**): High caregiver self-efficacy is associated with lower levels of caregiver burden and higher levels of caregiver gains; it mediates the indirect effect between caregiver burden and caregiver gains.

Other studies found that self-efficacy may not contribute to caregiver gains in the Chinese context [25]. This finding suggests that additional components that have not been considered in previous studies may exist, for example, social and cultural values.

### Authoritarian filial piety (AFP)

Instead of describing caregiving in terms of duty, responsibility, and burden, many family caregivers describe caregiving as a virtue and obligation. As aforementioned, filial piety embodies culture-specific Confucian ethics and moral principles concerning adult children’s attitudes and behaviours toward older parents. Empirical studies have confirmed that it has far-reaching implication for Chinese adults [32]. AFP underscores the elements of submission, conformity, and compliance. Individuals with high AFP tend to prioritize obeying their parents’ wishes owing to their seniority and authority. Thus, AFP may influence cognitive appraisals of caregiving, serving as a positive factor associated with lower burdens and acting as a buffer against role strain, while potentially contributing to negative outcomes, such as higher levels of depression, anxiety, and stress; therefore, detailed research is required to fully explore the role of AFP in the caregiving process. Consequently, we propose the following hypothesis:

- Hypothesis 2 (**H2**): Traditional AFP is associated with a higher level of caregiver burden and a lower level of caregiver gains; it moderates the association between caregiver burden, self-efficacy, and gains.

**Fig. 1:**
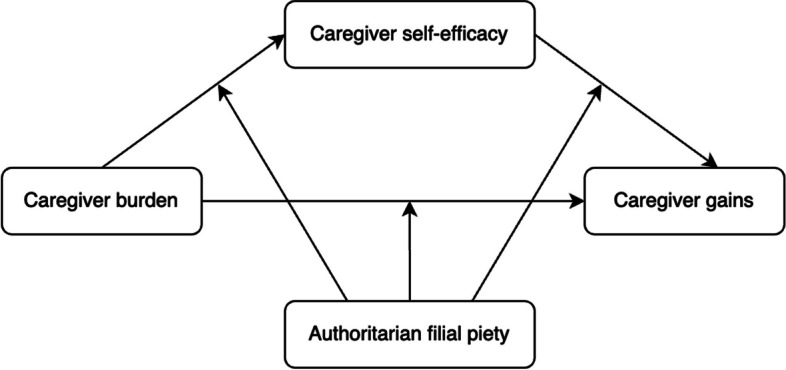
Proposed theoretical model

Based on the socio-cultural stress and coping model, the proposed theoretical model is presented in Fig. 1.

## Methods

### Data and sample

Data were collected from 1,854 adult caregiver/care-recipient dyads between July and August 2021 at local community eldercare centres from four major cities, in east China (Shanghai, Nanjing, Hangzhou, and Hefei). The inclusion criteria were as follows: respondents needed to be (1) the primary caregiver (aged 18–75 years old) caring for impaired older adults (≥ 60 years old) for more than 90 days during the past year; (2) provide a broad range of assistance (both ADL and IADL) for physically impaired elderly; (3) engaged in unpaid caregiving work. We excluded caregivers who cared for spouses and relatives, cognitively impaired older adults, and those with missing values in the analytic sample, resulting in 601adult children caregivers (age range: 20–74 years old)/ care-recipients (age range: 60–99 years old) dyads samples. A Human Ethics Approval Certificate was obtained from the author’s university before data collection commenced. For the selection of study participants, see Supplementary Figure S1.

### Variable measurement

#### Independent variable: caregiver burden

The key independent variable of interest in this study was caregiver burden. It was measured using a short version of the 4-item (screening) Zarit Burden Interview, for which the validity and reliability were confirmed by Bédard and Molloy [34]. The scale comprised four items rated on a 5-point scale. A higher mean ZBI score indicated a greater caregiver burden. In the present study, Cronbach’s α of the scale was 0.8063 (For details, see Supplementary Table S1).

#### Dependent variable: caregiver gains

The Chinese version of the PAC scale [13, 35] was used to measure caregiver gains. This scale focuses on positive gains during the caregiving process. The responses for each item are rated on a 5-point scale. A higher mean PAC score indicates greater caregiver gains. In the present study, Cronbach’s α of the scale was 0.6553 (For details, see Supplementary Table S2).

#### Mediating variable: caregiver self-efficacy

Caregiver self-efficacy was a mediating variable. This was assessed using a subscale of the Caregiver Task Inventory (CTI) developed and validated in the Chinese cultural context, which focuses on self-initiated actions and perceptions that serve to maintain or enhance the level of individual wellness, self-actualisation, and fulfilment reflected in five identified subscales [40]. The original CTI was lengthy for older family caregivers to complete; thus, a modified version containing six items was included. The responses for each item are rated on a 5-point scale. A higher mean CTI score indicates better caregiver self-efficacy. In the present study, Cronbach’s α of the scale was 0.8115 (Supplementary Table S3).

#### Moderating variable: AFP

AFP was the moderating variable in this study; it was measured using the six-item Measures for Family Values scale (6-item) from the 2006 China General Social Survey, and its validity and reliability have been confirmed in previous studies [41]. Assuming that the six items were equally important, we calculated the mean scores. A higher mean score indicates a traditional attitude toward AFP, which ranges from high to low. In the present study, Cronbach’s α of the scale was 0.7906 (Supplementary Table S4).

#### Control variables

The present study controlled for adult children caregivers’ gender (male = 1), age (continuous variable, ranging from 20–74 years old), marital status (married = 1), educational level (senior high school education = 1), employment status (employed = 1), and co-residence status with the care-recipient (not living together = 1). We also controlled for the care-recipient’s physical health status (categorical variable: 1 = mildly impaired, 2 = moderately impaired, 3 = severely impaired).

### Analytical plan

Four interlinked steps were used to test the hypotheses. First, we assessed the validity and reliability of each measurement scale. Second, Pearson’s correlation analysis and descriptive statistics were performed. Third, a moderated mediation model was employed using path analysis (a subset of structural equation modelling without latent variables) [42]. Based on the prior mean standardization of the scales, a simplified path analysis model is more aligned with the research objectives than SEM, as it requires a smaller sample size and is less sensitive to misspecifications [43]. Model 4 and Model 59 in PROCESS (a freely-available computational tool for SPSS and SAS developed by Andrew F. Hayes for mediation and moderation analysis in an integrated conditional process model) [44, 45] were applied to examine the mediating role of caregiver self-efficacy in the relationship between caregiver burden and caregiver gains, and the moderating effect of AFP on the above associations. Fourth, simple slope tests were used to determine whether the relationship between caregiver burden and caregiver gains varied for participants who scored 1 SD above and below the mean AFP level. SPSS 26.0 and Andrew Hayes SPSS process Macro 3.1 [45] were used to analyse the data.

## Results

### Sample characteristics

Table 1 shows the sample characteristics; 601 adult children caregiver/care-recipient dyads were selected. The proportions of mildly, moderately, and severely physically impaired care-recipients were 56.33%, 27.38%, and 16.29% respectively. A total of 62% of the adult children caregivers were women, with a mean age of 53.04 years (SD = 10.82). More than half (68%) had completed high school education, and 49% were employed. A total of 89% of the adult children caregivers were married, and the majority (75%) stated that they had been living with the care-recipients. The average caregiver burden (ZBI) and caregiver gains (PAC) were 2.32 (SD = 0.91) and 3.24 (SD = 0.77), respectively. Caregiver self-efficacy, the proposed mediator, averaged 2.82 (SD 0.6), and their AFP, the proposed moderator, averaged 2.15 (SD 0.53).

**Table 1:** Descriptive characteristics of the study sample (*N* = 601)

Variable	Mean (SD) or percentage
Gender	
Female	63.26%
Male	36.74%
Age (20–74 years)	53.05 (10.82)
Education	
Senior high school level	68.39%
Below senior high school level	31.61%
Marital status	
Married	88.83%
Unmarried	11.17%
Employment status	
Employed	48.83%
Unemployed	51.17%
Co-residence status with the care recipient	
Not living together	24.96%
Living together	75.04%
Caregiver burden (ZBI)	2.32(0.91)
Caregiver gains (PAC)	3.24(0.77)
Caregiver self-efficacy (CTI)	2.82 (0.6)
Authoritarian filial piety (AFP)	2.15(0.53)
Care recipients’ physical health status	
Mild impaired	56.33%
Moderate impaired	27.38%
Severe impaired	16.29%

### Caregiver self-efficacy as a mediator

We hypothesized that caregiver self-efficacy mediated the relationship between caregiver burden and caregiver gains. As shown in Table 2, the correlation coefficient between caregiver burden and caregiver gains was not significant (Beta = 0.039, *p* > 0.01). However, caregiver burden was negatively correlated with caregiver self-efficacy (Beta = -0.270, *p* < 0.001) and positively correlated with caregiver gains (Beta = 0.119, *p* < 0.001). This has supported H1, that caregiver self-efficacy mediated the relationship between caregiver burden and gains. We also conducted bootstrap tests for indirect, direct, and total effects (for details, please see Supplementary Table S5).

**Table 2:** Mediation results (*N* = 601)

Variables	Caregiver gains		Caregiver self-efficacy		Caregiver gains	
	**Beta**	**SE**	**Beta**	**SE**	**Beta**	**SE**
Caregiver burden	0.039	-0.04	-0.270***	-0.028	0.119***	-0.043
Caregiver self-efficacy					0.299***	-0.063
**Demographic Characteristics**						
Age	0.001	-0.004	0.003	-0.003	0.001	-0.004
Gender	0.052	-0.069	-0.01	-0.048	0.018	-0.069
Marital status	0.061	-0.107	0.118	-0.075	0.016	-0.107
Education	-0.014	-0.073	-0.07	-0.051	0.024	-0.073
Employment status	0.023	-0.082	0.055	-0.057	0.027	-0.081
Co-residence status	0.05	-0.076	0.138***	-0.053	0.035	-0.076
Health status (care-recipient)	0.071	-0.05	-0.087**	-0.035	0.091*	-0.05
Adjusted *R*^2^	-0.0027		0.2358		0.0361	

^*^P < 0.05; **P < 0.01; ***P < 0.001

### AFP as a moderator

**Fig. 2:**
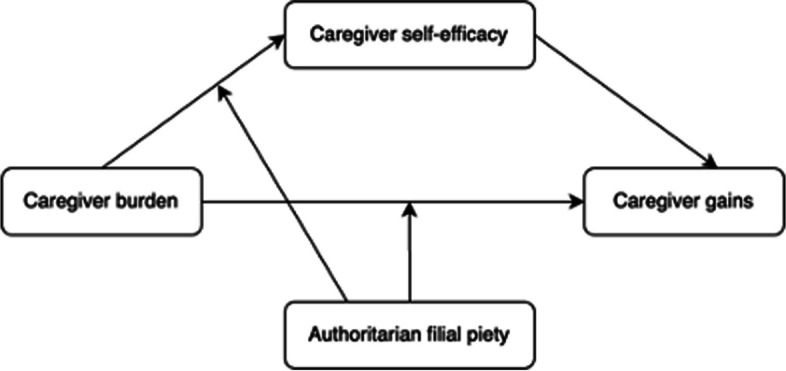
Model depiction of supported hypotheses

To test the second hypothesis, we examined the moderating effect of AFP on each portion of the indirect path within the mediation model (i.e., caregiver burden → caregiver self-efficacy, caregiver self-efficacy → caregiver gains, caregiver burden → caregiver self-efficacy → caregiver gains). No significant moderating effect of AFP was found on the pathway of “caregiver self-efficacy → caregiver gains”. As shown in Table 3, caregiver burden was negatively correlated with caregiver self-efficacy (Beta = -0.276, *p* < 0.001); the interaction of caregiver burden and AFP was also negatively correlated with caregiver self-efficacy (Beta = -0.077, *p* < 0.05), affirming the moderating effect of AFP on the pathway “caregiver burden → caregiver self-efficacy”. Meanwhile, the interaction of caregiver burden and AFP was also negatively correlated with caregiver gains (Beta = -0.199, *p* < 0.001). It indicated that AFP has a moderating effect on the pathway “caregiver burden → caregiver self-efficacy → caregiver gains”. Thus, H2 was partially supported (for verified pathways, please see Fig. 2, Figures S2 and S3 in supplementary materials).

**Table 3:** Mediation and moderated mediation results (*N* = 601)

Variables	Caregiver self-efficacy		Caregiver gains	
	**Beta**	**SE**	**Beta**	**SE**
Caregiver burden	-0.276***	-0.029	0.115***	-0.044
Caregiver self-efficacy			0.285***	-0.063
AFP	0.015	-0.045	0.032	-0.063
Caregiver burden × AFP	-0.077*	-0.044	-0.199***	-0.063
**Demographic Characteristics**				
Age	0.004	-0.003	0.002	-0.004
Gender	-0.014	-0.048	0.012	-0.069
Marital status	0.11	-0.075	0.005	-0.107
Education	-0.059	-0.052	0.013	-0.074
Employment status	0.053	-0.058	0.028	-0.082
Co-residence status	0.138**	-0.054	0.061	-0.077
Health status(care-recipient)	-0.078**	-0.035	0.098*	-0.05
Adjusted *R*^2^	0.2086		0.0498	

*AFP* Authoritarian Filial Piety

^*^P < 0.05; **P < 0.01; ***P < 0.001

**Fig. 3:**
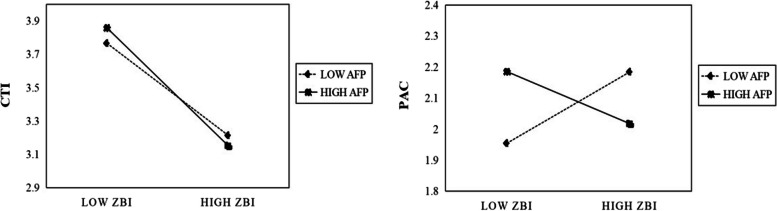
Test of moderation model. PAC. Note: PAC—Positive Aspects of Caregiving (caregiver gains), CTI—Caregiver Task Inventory (caregiver self-efficacy), AFP—Authoritarian Filial Piety. Total *N* = 601

To further visualize these effects and pathways, two simple slopes were plotted. Figure 3 illustrates a buffering effect of AFP on the pathway of “caregiver burden → caregiver self-efficacy”. The slope was steeper for participants who reported a high AFP attitude, indicating that AFP strengthened the negative correlation between caregiver burden and caregiver self-efficacy. Moreover, in the “caregiver burden → caregiver self-efficacy → caregiver gains” pathway, the slopes exhibited opposite directions, signifying that AFP attenuated or mitigated the positive correlation between caregiver burden and caregiver gains.

## Discussion

Using cross-sectional data of Chinese adult children caregivers caring for physically impaired older parents, this is the first study to provide initial insights into the mediating role of caregiver self-efficacy and the moderating role of the underpinning cultural value AFP in the caregiving process. Based on the socio-cultural coping and stress model, this study contributes to the caregiving literature by identifying two striking findings.

### Caregiver self-efficacy transforms caregiver burden into caregiver gains

Consistent with the caregiver adaptation model and previous studies showing that high positive gains are most likely when caregivers with high self-efficacy face high care-recipient demands over the long term [22, 26, 27, 39], our findings revealed that caregiver self-efficacy not only mediates the association between caregiver burden and caregiver gains among Chinese caregivers, but may also transform caregiver burden into caregiver gains. That is, enhancing caregiver self-efficacy significantly fosters better caregiving capacity in managing stress and achieving a sense of gain.

### AFP function has a negative effect on caregiver gains

Our finding highlights the negative effect of AFP on caregiving experiences. Moderated mediation analyses suggested that AFP mediates both the significant association between “caregiver burden and caregiver self-efficacy” and “caregiver burden and caregiver gains”. This indicates that adult children caregivers endorsing AFP may have lower caregiver self-efficacy, which prevents them from finding a sense of gain in their caregiving experiences. Meanwhile, our findings are in line with existing findings that adults who adhere to AFP may encounter challenges in effectively managing their parents’ limitations and behaviours, attributed to an over-emphasis on the hierarchical aspects of family [31]. Chinese adult children who adhere to AFP may lean toward emotional-focused coping (i.e., self-sacrifice, self-blame, obedience) rather than adopting solution-focused coping (i.e., utilization of formal support services and respite services, accept help from others), resulting in a subjectively more challenging caregiving journey for their older parents.

### Implications

Derived from the concept of intergenerational reciprocity within the Chinese family structure, performing filial piety is obligatory for Chinese individuals to maintain psychological homeostasis. Traditional AFP attitude may regulate intergenerational caregiving to guarantee family support but may also lead to less intimate and more obedient relationships, preventing utilization of formal support, seeking help, and further decreasing positive gains in caregiving. Our findings provide valuable insights for interventions for adult children caregivers in ageing societies with similar cultural norms. To sustain traditional caregiving practices, in addition to providing practical coping skills and intervention programmes to improve caregiver self-efficacy, culturally sensitive psychoeducational therapy should be provided to alleviate feelings of the potential subjective burden arising from cultural expectations.

### Limitations and future studies

This study has certain limitations. First, it is difficult to infer causality using a cross-sectional design. Future longitudinal and nationally representative samples are required to further explore the role of AFP in different caregiving stages. Second, the present study only tested the role of AFP in the caregiving process; thus, to gain a complete picture of the role of filial piety, future research should explore the effects of both RFP and AFP on caregiving outcomes. Third, this study measured family caregivers’ self-efficacy and caregiver gains through a subscale based on the Chinese context; thus, a comparison of the present findings with those based on other scales should be treated with caution. Future studies should use international scales to generate comparable results across cultures. Despite these limitations, this study is among the first to comprehensively examine ways AFP shapes a family caregiver’s experience, together with caregiver self-efficacy.

## Conclusion

Using cross-sectional data from 601 adult children caregiver/care-recipient dyads from four capital cities in China, our study found that AFP may hinder caregiver self-efficacy and impede positive gains in the caregiving process. Culturally sensitive psychoeducational therapy that assists in managing AFP expectations and enhances caregiver self-efficacy can be considered an integral intervention to improve positive gains in caregiving.

### Supplementary Information

**Additional file 1: Figure S1.** The selection of study participants. **Figure S1.** The selection of study participants. **Figure S2.** Conceptual and statistical representation of the exploratory analysis depicting moderated moderation of an indirect effect (see Table 2 for estimates). **Figure S3.** Conceptual and statistical representation of the exploratory analysis depicting moderated moderation of an indirect effect (see Table 3 for estimates). **Table S1.** Caregiver scores on Caregiver Burden (ZBI). **Table S2.** Caregiver scores on Positive Aspects of caregiving (PAC). **Table S3.** Caregiver scores on Caregiver Task Inventory (CTI). **Table S4.** Caregiver scores on Authoritarian Filial Piety (AFP). **Table S5.** Bootstrap test of the mediator of CTI.

## Data Availability

The datasets generated and/or analysed during the current study are not publicly available owing to privacy reasons but are available from the corresponding author on reasonable request.

## Abbreviations

RFP: Reciprocal filial piety
AFP: Authoritarian filial piety

## References

[1] Pristavec T, Pruchno R (2019). The burden and benefits of caregiving: a latent class analysis. Gerontologist.

[2] Lai DWL (2007). Cultural predictors of caregiving burden of Chinese-Canadian family caregivers. Can J Aging.

[3] Sherwood PR, Given CW, Given BA, Von Eye A (2005). Caregiver burden and depressive symptoms: analysis of common outcomes in caregivers of elderly patients. J Aging Health.

[4] Xu L, Liu Y, He H, Fields NL, Ivey DL, Kan C (2021). Caregiving intensity and caregiver burden among caregivers of people with dementia: the moderating roles of social support. Arch Gerontol Geriatr.

[5] Definitions - Family Caregiver Alliance. https://www.caregiver.org/resource/definitions-0/. Accessed 4 Nov 2023.

[6] Stajduhar KI, Funk L, Toye C, Grande G, Aoun S, Todd C (2010). Part 1: Home-based family caregiving at the end of life: a comprehensive review of published quantitative research (1998–2008). Palliat Med.

[7] Hsiao CY (2010). Family demands, social support and caregiver burden in Taiwanese family caregivers living with mental illness: the role of family caregiver gender. J Clin Nurs.

[8] Park M, Choi S, Lee SJ, Kim SH, Kim J, Go Y (2018). The roles of unmet needs and formal support in the caregiving satisfaction and caregiving burden of family caregivers for persons with dementia. Int Psychogeriatr.

[9] Chan CK, Vickers T, Barnard A (2021). Meaning through caregiving: a qualitative study of the experiences of informal carers. Br J Soc Work.

[10] MacKenzie A, Greenwood N (2021). Positive experiences of caregiving in stroke: a systematic review. Disabil Rehabil.

[11] Lawton MP (2021). Personal values profiles in dementia family caregivers: their association with ambivalent feelings and anxious and depressive symptoms. Aging Ment Health.

[12] Pendergrass A, Mittelman M, Graessel E, Özbe D, Karg N (2019). Predictors of the personal benefits and positive aspects of informal caregiving. Aging Ment Health.

[13] Lou VWQ, Lau BHP, Cheung KSL (2015). Positive aspects of caregiving (PAC): scale validation among Chinese dementia caregivers (CG). Arch Gerontol Geriatr.

[14] Yu DSF, Cheng ST, Wang J (2018). Unravelling positive aspects of caregiving in dementia: an integrative review of research literature. Int J Nurs Stud.

[15] Steffen AM, McKibbin C, Zeiss AM, Gallagher-Thompson D, Bandura A (2002). The revised scale for caregiving self-efficacy: reliability and validity studies. J Gerontol B Psychol Sci Soc Sci.

[16] Smaling HJA, Joling KJ, Achterberg WP, Francke AL, van der Steen JT (2021). Measuring positive caregiving experiences in family caregivers of nursing home residents: a comparison of the Positive Experiences Scale, Gain in Alzheimer Care INstrument, and Positive Aspects of Caregiving questionnaire. Geriatr Gerontol Int.

[17] Pharr JR, Dodge Francis C, Terry C, Clark MC (2014). Culture, caregiving, and health: exploring the influence of culture on family caregiver experiences. ISRN Public Health.

[18] Knight BG, Sayegh P (2010). Cultural values and caregiving: the updated sociocultural stress and coping model. J Gerontol B Psychol Sci Soc Sci.

[19] Lai DWL (2010). Filial piety, caregiving appraisal, and caregiving Burden. Res Aging.

[20] Aranda MP (1997). The influence of ethnicity and culture on the caregiver stress and coping process: a sociocultural review and analysis. Gerontologist.

[21] Kramer BJ (1997). Gain in the caregiving experience: where are we? What next?. Gerontologist.

[22] Lawton MP, Moss M, Kleban MH, Glicksman A, Rovine M (1991). A two-factor model of caregiving appraisal and psychological well-being. J Gerontol.

[23] Pristavec T, Pruchno R (2019). The burden and benefits of caregiving: a latent class analysis. Gerontologist.

[24] Pakenham KI, Cox S (2008). Development of the benefit finding in multiple sclerosis (MS) caregiving scale: a longitudinal study of relations between benefit finding and adjustment. Br J Health Psychol.

[25] Yan Z, Zhang J, Sun X. Burdened but meaningful?: How gender role attitudes influence the complex links between care-giver self-efficacy, formal support utilisation and benefit-finding among spousal care-givers. Br J Soc Work. 2023;1–23. 10.1093/bjsw/bcad184.

[26] Chenoweth L, Stein-Parbury J, White D, McNeill G, Jeon Y-H, Zaratan B (2016). Coaching in self-efficacy improves care responses, health and well-being in dementia carers: a pre/post-test/follow-up study. BMC Health Serv Res.

[27] Yang Z, Tian Y, Fan Y, Liu L, Luo Y, Zhou L (2019). The mediating roles of caregiver social support and self-efficacy on caregiver burden in Parkinson’s disease. J Affect Disord.

[28] Losada-Baltar A, Vara-García C, Pedroso-Chaparro MDS, Cabrera I, Jiménez-Gonzalo L, Fernandes-Pires J, Huertas-Domingo C, Barrera-Caballero S, Gallego-Alberto L, Romero-Moreno R, Márquez-González M. Family caregivers of people with dementia in the context of the sociocultural stress and coping model: An examination of gender differences. J Women Aging. 2023;35(4):354–68. 10.1080/08952841.2022.2052705 35343403

[29] Bedford O, Yeh KH (2021). Evolution of the conceptualization of filial piety in the global context: from skin to skeleton. Front Psychol.

[30] Khalaila R, Litwin H (2011). Does filial piety decrease depression among family caregivers?. Aging Ment Health.

[31] Wu CW, Yeh KH (2021). Self-sacrifice is not the only way to practice filial piety for Chinese adolescents in conflict with their parents. Front Psychol..

[32] Jen CH, Chen WW, Wu CW (2019). Flexible mindset in the family: filial piety, cognitive flexibility, and general mental health. J Soc Pers Relat.

[33] Liu Z, Heffernan C, Tan J (2020). Caregiver burden: a concept analysis. Int J Nurs Sci.

[34] Bédard M, Molloy DW, Squire L, Dubois S, Lever JA, O’Donnell M (2001). The Zarit Burden Interview: a new short version and screening version. Gerontologist.

[35] Yu DSF, Cheng ST, Wang J (2018). Unravelling positive aspects of caregiving in dementia: An integrative review of research literature. Int J Nurs Stud.

[36] Bandura A (1978). Self-efficacy: toward a unifying theory of behavioral change. Adv Behav Res Ther.

[37] Schumacher KL, Stewart BJ, Archbold PG (1998). Conceptualization and measurement of doing family caregiving well. J Nurs Scholarsh.

[38] Roth DL, Fredman L, Haley WE (2015). Informal caregiving and its impact on health: a reappraisal from population-based studies. Gerontologist.

[39] Cheng ST, Lam LCW, Kwok T, Ng NSS, Fung AWT (2013). Self-efficacy is associated with less burden and more gains from behavioral problems of Alzheimer’s disease in Hong Kong Chinese caregivers. Gerontologist.

[40] Lee RLT, Mok ESB (2011). Evaluation of the psychometric properties of a modified Chinese version of the Caregiver Task Inventory - refinement and psychometric testing of the Chinese Caregiver Task Inventory: a confirmatory factor analysis. J Clin Nurs.

[41] Hu Y, Scott J (2014). Family and gender values in China: generational, geographic, and gender differences. J Fam Issues.

[42] Structural Equation Modeling (SEM). n.d. http://faculty.cas.usf.edu/mbrannick/regression/SEM.html. Accessed 04 Nov 2023.

[43] Devlieger I, Rosseel Y (2017). Factor score path analysis: an alternative for SEM?. Methodology.

[44] Hayes AF. PROCESS: a versatile computational tool for observed variable moderation, mediation, and conditional process modeling. [White paper]. Retrieved from 2012;1–39. http://www.afhayes.com/public/process2012.pdf.

[45] Hayes AF. Introduction to mediation, moderation, and Ccnditional process analysis: a regression-based approach. New York: Guilford Press; 2017.

