# Supplementary material for: Blessing or curse: the role of authoritarian filial piety and self-efficacy in caregiver gains among Chinese family caregivers caring for physically impaired older adults
Source: BMC Geriatr. 2024 Feb 16;24:163. doi: 10.1186/s12877-024-04768-x (PMC10870663; doi:10.1186/s12877-024-04768-x)
Supplement: Supplementary file 1 — Additional file 1: Figure S1. The selection of study participants. Figure S1. The selection of study participants. Figure S2. Conceptual and statistical representation of the exploratory analysis depicting moderated moderation of an indirect effect (see Table 2 for estimates). Figure S3. Conceptual and statistical representation of the exploratory analysis depicting moderated moderation of an indirect effect (see Table 3 for estimates). Table S1. Caregiver scores on Caregiver Burden (ZBI). Table S2. Caregiver scores on Positive Aspects of caregiving (PAC). Table S3. Caregiver scores on Caregiver Task Inventory (CTI). Table S4. Caregiver scores on Authoritarian Filial Piety (AFP). Table S5. Bootstrap test of the mediator of CTI. [file 12877_2024_4768_MOESM1_ESM.docx]

Supplementary material


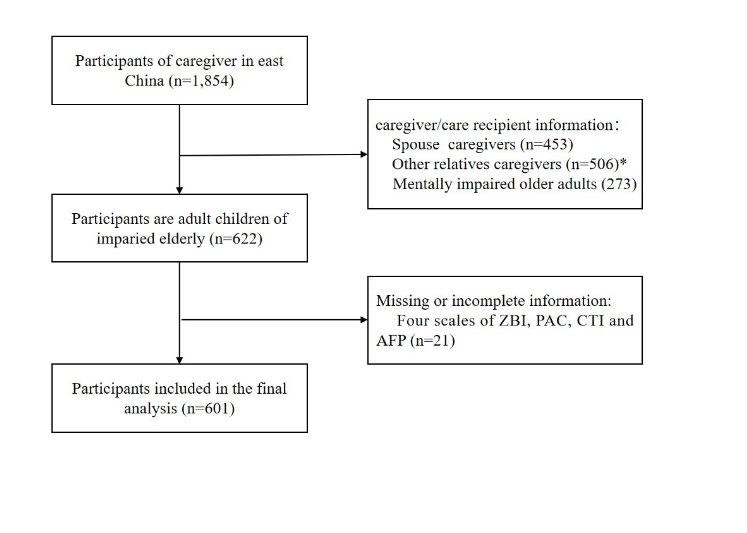


Figure S1 The selection of study participants


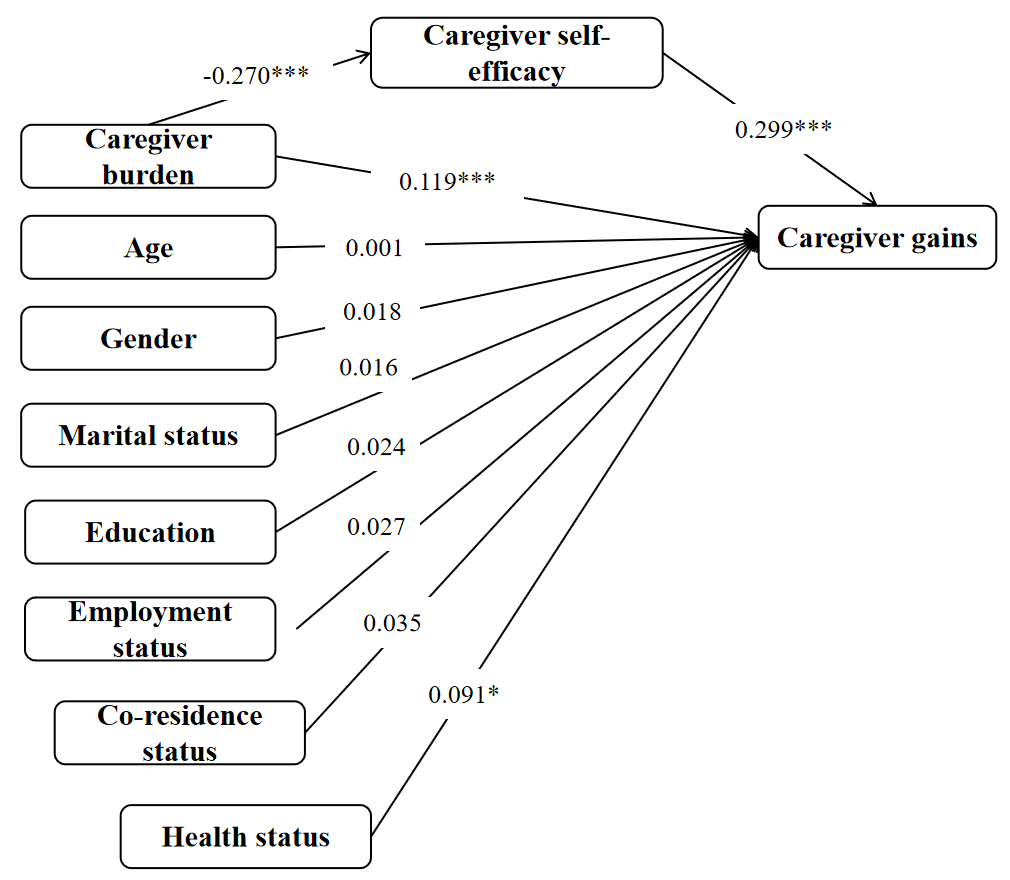


Figure S2 Conceptual and statistical representation of the exploratory analysis depicting moderated moderation of an indirect effect (see Table 2 for estimates)


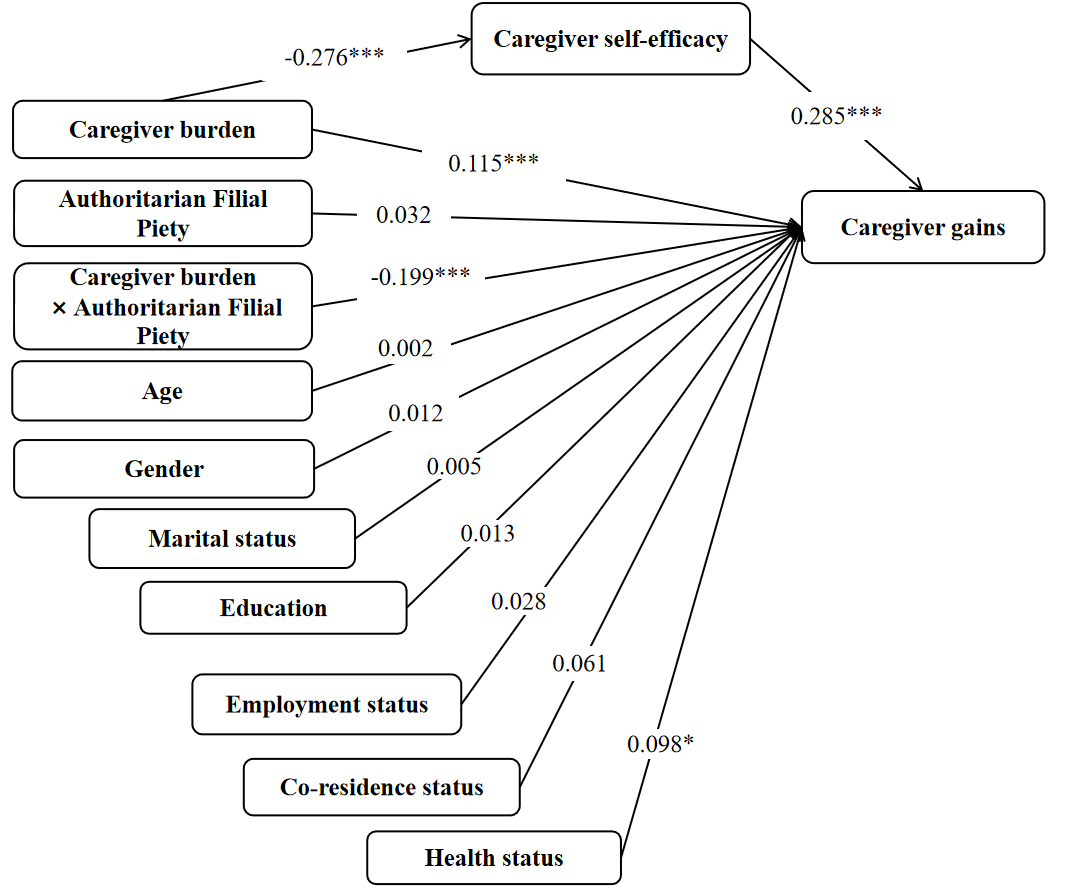


Figure S3 Conceptual and statistical representation of the exploratory analysis depicting moderated moderation of an indirect effect (see Table 3 for estimates)

Table S1 Caregiver scores on Caregiver Burden (ZBI)

| Items | Measures |
| --- | --- |
| Role strain | Because of the time you spend with your relative that you don’t have enough time for yourself? |
| Psychological well-being | Do you feel strained when you are around your relatives? |
| Career development | Do you feel stressed between caring for your relative and trying to meet other responsibilities (work/family)? |
| Physical health | Do you have impaired motor capacity due to caring activities? |
| Cronbach’s α = 0.8063 | |

Source: Bédard, M., Molloy, D. W., Squire, L., Dubois, S., Lever, J. A., O'Donnell, M. (2001). The Zarit Burden Interview: A New Short and Screening Version. Gerontology 41(5), 652-657.

Table S2 Caregiver scores on Positive Aspects of caregiving (PAC)

| Items | Measures |
| --- | --- |
| Role satisfaction | Providing help to the care receiver has made me feel appreciated. |
| Emotional rewards | Providing help to the care receiver has made me appreciate life more. |
| Relationship gains | Providing help to the care receiver strengthened my relationship with others. |
| Cronbach’s α =0.6553 | |

Source: Lou, V. W. Q., Lau, B. H. P. & Cheung, K. S. L. Positive aspects of caregiving (PAC): Scale validation among Chinese dementia caregivers (CG). Arch Gerontol Geriatr 60, 299–306 (2015)

Table S3 Caregiver scores on Caregiver Task Inventory (CTI)

| Items | Measures |
| --- | --- |
| Gain knowledge about the disease. | Do you have difficulty increasing your knowledge about the disease and caring? |
| Learning to cope with a new role: involves attending to and accepting responsibility for one’s ability to cope with new tasks in caregiving. | Do you have difficulty performing basic ADL for the care receiver? |
| Providing care with the care receiver’s needs in mind involves establishing patterns and routines to meet the care receiver’s daily living activities. | Do you have difficulty supervising prescribed treatments and general recommendations? |
| Managing one’s own emotional needs involves dealing with personal feelings towards the care-receiver and caregiving. | Do you have difficulty emotionally accepting the likelihood of a progressive downward course? |
| Appraising supportive resources involves reflecting satisfaction with the support received and with the number and composition of one’s interpersonal ties. | Do you have difficulty balancing the giving of assistance with responsibilities to other family members? |
| Balancing caregiving needs and one’s own needs involves attaining self-actualization and fulfilment. | Do you have difficulty readjusting your routine? |
| Cronbach’s α =0.8115 | |

Source: Lee R. L. and Mok (2011). Evaluation of the psychometric properties of a modified Chinese version of the Caregiver Task Inventory–Refinement and psychometric testing of the Chinese Caregiver Task Inventory: A confirmatory factor analysis. Journal of Clinical Nursing, 20(23–24), 3452–3462.

Table S4 Caregiver scores on Authoritarian Filial Piety (AFP)

| Items | Measures |
| --- | --- |
| Jing (nonmaterial filial piety) | One should treat their parents kindly regardless of how one has been treated. |
|  | One should honour their parents and make them proud. |
| Xiao (material filial piety) | One should follow parents’ choice and give up their wish. |
|  | One should live with parents after getting married. |
|  | One should constantly go home to visit parents, even if working in other places. |
| patrilineality | To preserve the family lineage, one should give birth to at least one male heir. |
| Cronbach’s α =0.7906 | |

Source: China General Social Survey, 2006.

Table S5 Bootstrap test of the mediator of CTI

|  | effect value | SE | 95% Conf. Interval | | proportion effect value |
| --- | --- | --- | --- | --- | --- |
| indirect effect | -0.079 | 0.024 | -0.132 | -0.037 | -2.03 |
| direct effect | 0.119 | 0.043 | 0.032 | 0.207 | 3.05 |
| total effect | 0.039 | 0.043 | 0.034 | 0.204 |  |

Note: CTI: Caregiver Task Inventory, Total n = 601.
